# High-throughput screening of circRNAs reveals novel mechanisms of tuberous sclerosis complex-related renal angiomyolipoma

**DOI:** 10.1186/s40246-021-00344-1

**Published:** 2021-07-09

**Authors:** Yang Zhao, Hao Guo, Wenda Wang, Guoyang Zheng, Zhan Wang, Xu Wang, Yushi Zhang

**Affiliations:** 1 Department of Urology, Peking Union Medical College Hospital, Chinese Academy of Medical Sciences and Peking Union Medical College, Beijing, China; 2 Department of Urology, Chengdu Second People’s Hospital, Chengdu, Sichuan China

**Keywords:** Tuberous sclerosis, Renal angiomyolipoma, Circular RNAs

## Abstract

**Objective:**

Tuberous sclerosis complex (TSC) is a rare autosomal dominant disease characterized by lesions throughout the body. Our previous study showed the abnormal up-regulation of miRNAs plays an important part in the pathogenesis of TSC-related renal angiomyolipoma (TSC-RAML). circRNAs were known as important regulators of miRNA, but little is known about the circRNAs in TSC-RAMLs.

**Methods:**

Microarray chips and RNA sequencing were used to identify the circRNAs and mRNAs that were differently expressed between the TSC-RAML and normal kidney tissue. A competitive endogenous RNA (ceRNA) regulatory network was constructed to reveal the regulation of miRNAs and mRNAs by the circRNAs. The biological functions of circRNA and mRNA were analyzed by pathway analysis. Microenvironmental cell types were estimated with the MCP-counter package.

**Results:**

We identified 491 differentially expressed circRNAs (DECs) and 212 differentially expressed genes (DEGs), and 6 DECs were further confirmed by q-PCR. A ceRNA regulatory network which included 6 DECs, 5 miRNAs, and 63 mRNAs was established. Lipid biosynthetic process was significantly up-regulated in TSC-RAML, and the humoral immune response and the leukocyte chemotaxis pathway were found to be down-regulated. Fibroblasts are enriched in TSC-RAML, and the up-regulation of circRNA_000799 and circRNA_025332 may be significantly correlated to the infiltration of the fibroblasts.

**Conclusion:**

circRNAs may regulate the lipid metabolism of TSC-RAML by regulation of the miRNAs. Fibroblasts are enriched in TSC-RAMLs, and the population of fibroblast may be related to the alteration of circRNAs of TSC-RAML. Lipid metabolism in fibroblasts is a potential treatment target for TSC-RAML.

**Supplementary Information:**

The online version contains supplementary material available at 10.1186/s40246-021-00344-1.

## Introduction

Tuberous sclerosis complex (TSC) is a rare autosomal dominant disease characterized by lesions throughout the body. The incidence of TSC is estimated to be 1/6000 to 1/10,000 in live births and around 1/20,000 in population [1, 2]. TSC-related renal angiomyolipoma (TSC-RAML) can occur in 70–90% TSC patients, and it is the leading cause of mortality in male adult TSC patients [3]. Most TSC-RAMLs are bilateral multiple kidney tumors [4]. With the progression of TSC-RAMLs, TSC patients may suffer from chronic renal insufficiency or even renal failure [5].

Mutations in TSC1 or TSC2 gene, which encodes the tuberin and hamartin, could be found in 75–90% TSC patients, while a significant fraction (10–25%) of TSC patients have no mutation identified by conventional genetic testing [2]. The mutation in TSC1 or TSC2 gene could cause the inactivation of the tuberin or hamartin protein, which further lead to the over-activation of mTOR signaling pathway. mTOR inhibitors were used as the major treatment for TSC-RAML [6, 7]. EXISTII trial showed that 58% of the TSC-RAML patients reached 50% reduction in tumor size after receiving everolimus treatment for 1 year. However, 21% of these patients have poor response to everolimus treatment, and 14.3% of the TSC-RAMLs even showed tumor progression. Further knowledge of the pathogenesis of TSC-RAML is needed in order to find new treatment targets of the TSC-RAML.

Non-coding RNAs are known to be important regulators of cell behavior. Abnormal non-coding RNA expression was found to be essential in the initiation and progression in various malignant and benign human tumors [8]. In our previous study, we also found the abnormal up-regulation of miR-9-5p, miR-124-3p, and miR-132-3p in TSC-RAML tissue and proved that these miRNAs could regulate the proliferation and apoptosis of TSC2-deficient fibroblasts [9]. But little is known about the regulation of miRNAs in the TSC-RAML.

Circular RNAs (circRNAs) are recently identified as a novel group of non-coding RNAs. The closed-loop structure of circRNAs could cause high stability and abundance of circRNA in cell plasma and body fluid [10, 11]. circRNAs could regulate the expression level of the downstream mRNAs by functioning as sponges of miRNAs [11]. Increasing evidence showed that circRNAs were involved in the tumorigenesis and progression of human tumors, such as renal cell carcinoma [8]. So far, however, there is no study about the expression of circRNAs in TSC-RAMLs. In this study, we explored the expression of circRNAs and matched transcriptome in TSC-RAML using microarray chips and transcriptome sequencing, aiming to shine new light on the regulation of non-coding RNA in TSC-RAMLs.

## Materials and methods

### Patients and specimens

TSC diagnosis was based on the 2012 International Tuberous Sclerosis Complex Consensus Conference [12]. Patients with 2 major TSC criteria or 1 major and 2 minor TSC criteria were included. TSC-RAML samples were acquired from four TSC patients during surgery, and normal kidney tissues from three patients with other benign kidney diseases were used as normal controls. The detailed clinical information of all patients is listed in table S1. Specimens were stored at − 80 °C until total RNA extraction. All TSC-RAML and normal kidney specimens were validated by pathology examination.

This study protocol was approved by the Human Ethics Committee of Peking Union Medical College Hospital. Informed consent was obtained from all patients included in the study. The procedures for the collection and use of tissues were performed in accordance with the ethical standards set forth in the Declaration of Helsinki.

### Microarray analysis and differentially expressed circRNA (DEC) screening

Total RNAs were digested with Rnase R (Epicentre, Inc.) to remove linear RNAs and enrich circular RNAs. Then, the enriched circular RNAs were amplified and transcribed into fluorescent cRNA utilizing a random priming method (Arraystar Super RNA Labeling Kit; Arraystar). The labeled cRNAs were hybridized onto the Arraystar Human circRNA Array V2 (8x15K, Arraystar). The slides were then washed and scanned by the Agilent Scanner G2505C.

Agilent Feature Extraction software (version 11.0.1.1) was used to analyze acquired array images. Quantile normalization and subsequent data processing were performed using the limma package of the R software. Clustering analysis was performed using the hclust function of the R software. Differentially expressed circRNAs with statistical significance between two groups were identified. Quantile normalization and subsequent data processing were performed using the R software limma package. Differentially expressed circRNAs with statistical significance between two groups were identified using the limma package. circRNAs with |fold change| > 2 and FDR < 0.01 were recognized as differentially expressed circRNAs (DECs).

### Quantitative real-time PCR

Total RNA from cell lines was extracted using Trizol solution (Invitrogen, USA). The quantities and qualities of isolated RNAs were evaluated by the NanoDrop ND-1000 (Thermo Fisher Scientific, USA). Total RNAs were converted into cDNA using SuperScript III Reverse Transcriptase (Invitrogen, USA). RT-PCR was performed using 2X PCR master mix (Arraystar, USA) with the following program: initial denature at 95 °C for 10 min, followed by 40 cycles of 95 °C for 10 s and 60 °C for 60 s. β-actin was used as a control. Three independent replicates were performed for each group. The sequences of primers were listed in table S2. Student’s t test was used to evaluate the difference of circRNA between the TSC-RAML and the normal controls.

### Transcriptome sequencing and differentially expressed gene (DEG) screening

Total RNAs were extracted from samples and the quantity of total RNAs was measured by the NanoDrop ND-1000 (Thermo Fisher Scientific, USA). Ribosomal RNA was removed using RiboZero Magnetic Gold Kit (Epicentre, USA) and then a library was constructed using KAPA Stranded RNA-Seq Library Prep Kit (Illumina, USA). Then, the integrity of the library was measured by the Agilent Bioanalyzer 2100 (Agilent Technologies, USA). High-throughput RNA sequencing was performed with Illumina HiSeq 4000 sequencer. The clean reads were aligned to the reference genome by Hisat2.

Raw read counts were then normalized using the TPM method. Differentially expressed mRNAs with statistical significance between two groups were identified using the limma package. The mRNAs with |fold change| > 2 and FDR < 0.01 were recognized as differentially expressed genes (DEGs).

Gene-set variant analysis (GSVA) analysis was performed using the GSVA package (v 1.38.1) of R software to detect the alteration of mTOR pathway [13]. The mTOR gene sets used in the GSVA analysis were downloaded from the Molecular Signatures Database (MSigDB, https://www.gsea-msigdb.org) [14]. The result of the GSVA analysis was shown in the histogram.

### Previously published data and robust rank aggregation (RRA) analysis

We have previously detected mRNA expression matrix of four TSC-RAML samples using microarray chips [9]. The DEGs from our previous publication were retrieved, and the overlap of DEGs from the two studies was displayed with a Venn diagram. RankAggreg package of R software was used for DEG list integration [15].

### Pathway analysis

Pathway analysis was conducted using the Metascape software (https://metascape.org) [16]. DEGs were mapped to pathway terms including the KEGG Pathway, Hallmark Gene Sets, Canonical Pathways, BioCarta Gene Sets, GO Biological Processes, and Reactome Gene Sets, and the top 10 enriched terms, which were determined by the P value, were presented in using dot plot.

### Estimation of microenvironmental cell type

Based on our transcriptome sequencing data, the infiltration of fibroblasts, endothelial cells, and immune cells were estimated by the MCP-counter package of R software [17]. Pearson’s correlation analysis was performed to analyze the relationship between the DECs and the population of these cells.

### Establishment of ceRNA regulatory network

In order to demonstrate the regulatory relationships among circRNAs, miRNAs, and mRNAs, DECs and DEGs identified in this study and differentially expressed microRNAs reported in our previous publications [9] were used to construct a ceRNA regulatory network. miRwalk (http://mirwalk.umm.uni-heidelberg.de) was used to predict miRNA binding sites, and the ceRNA network which consisted of circRNA-miRNA pairs and miRNA-mRNA pairs with the same miRNA nodes was visualized by Cytoscape 3.7.1.

### Statistical analysis

Statistical analysis was performed using R software. Unless specifically mentioned, P < 0.05 was considered to be statistically significant.

## Results

### Differentially expressed circRNA and mRNA profile by sequencing

**Fig. 1:**
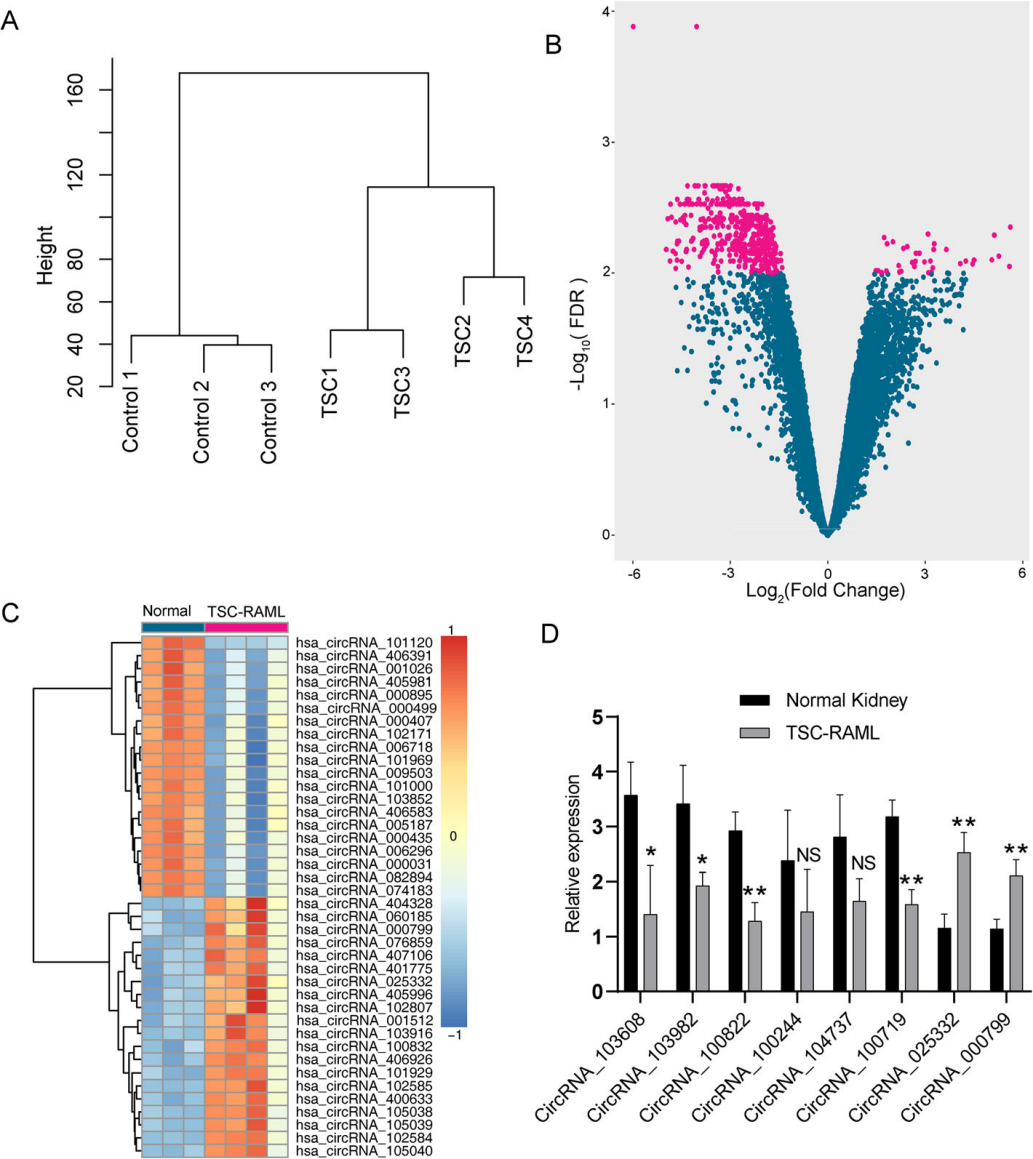
Differentially expressed circRNA profile identified by microarray chips. **A** Clustering dendrogram of the circRNA data. **B** The volcano plot showing change of all circRNAs between TSC-RAML and normal kidney tissue. Each pink dot stands for one DEC. The x-axis indicates the log2-transformed fold change, and the y-axis refers to the -log10-transformed false discovery rate (FDR). **C** Cluster heatmap showing the expression of the top 20 up-regulated DECs and the top 20 down-regulated DECs. **D** The relative expression of 8 DECs were tested using q-PCR. *P < 0.05; **P < 0.01

circRNA profiles of TSC-RAML and the normal kidney were detected using microarray chips. Clustering analysis showed that the difference of circRNAs is small inside the two groups and big between the two groups (Fig. 1A). Thirty-five up-regulated DECs and 456 down-regulated DECs were identified (Fig. 1B, C).

**Fig. 2:**
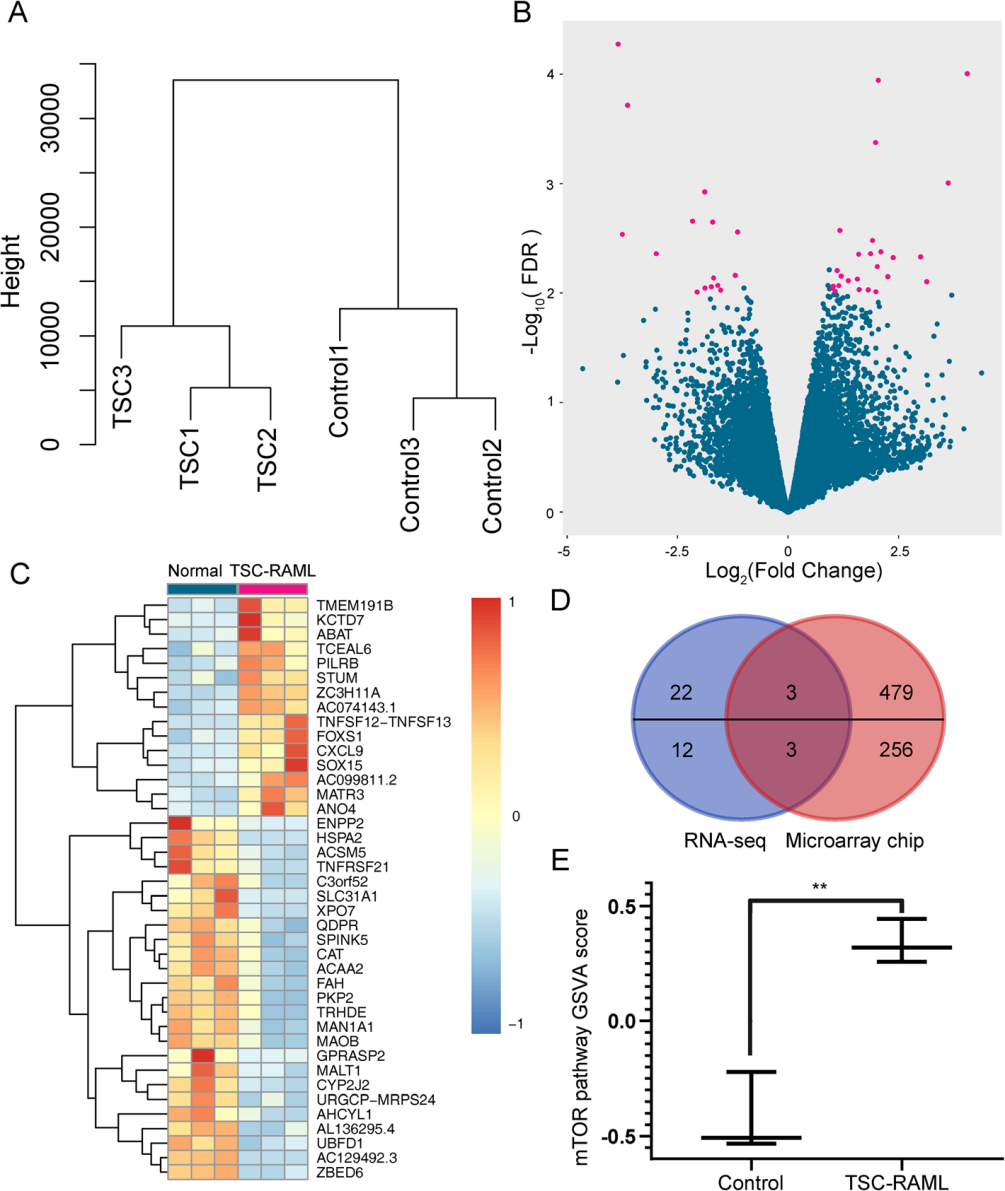
Differentially expressed mRNA profile identified by sequencing. **A** Clustering dendrogram of the mRNA data. **B** The volcano plot showing change of all DEGs between TSC-RAML and normal kidney tissue. Each pink dot stands for one DEG. The x-axis indicates the log2-transformed fold change, and the y-axis refers to the -log10-transformed false discovery rate (FDR). **C** Cluster heatmap showing the expression of all DEGs.**D** Venn diagram showing the overlap of DEGs between the current study and our previous microarray mRNA chip study. Six genes were found to be overlap DEGs between the two studies, including MALT1, UBFD1, AMACR (up-regulated DEGs), KCNK3, ABAT, and STUM (down-regulated DEGs). **E** GSVA analysis of the mTOR pathway. The TSC-RAML group have higher mTOR GSVA scores than the control group (P = 0.0026, **E**)

The matched mRNA profiles were detected using transcriptome sequencing, and one TSC-RAML sample was excluded for insufficient samples. Clustering analysis showed that the difference of mRNAs is big between the two groups (Fig. 2A). Twenty-five up-regulated genes and 15 down-regulated genes were found (Fig. 2B, C). We have previously detected the mRNA expression matrix of four TSC-RAML samples using microarray chips, and the DEGs from our previous publication were retrieved. Six genes were found to be overlap DEGs between the two studies, including MALT1, UBFD1, AMACR (up-regulated DEGs), KCNK3, ABAT, and STUM (down-regulated DEGs) (Fig. 2D). RRA package was used for DEG list integration, and 98 up-regulated DEGs and 114 down-regulated DEGs were identified after the integration. GSVA analysis showed that the TSC-RAML group have higher mTOR GSVA scores than the control group, which is consistent with the mechanism of TSC pathogenesis (P = 0.0026, Fig. 2E).

### Pathway analysis

**Fig. 3:**
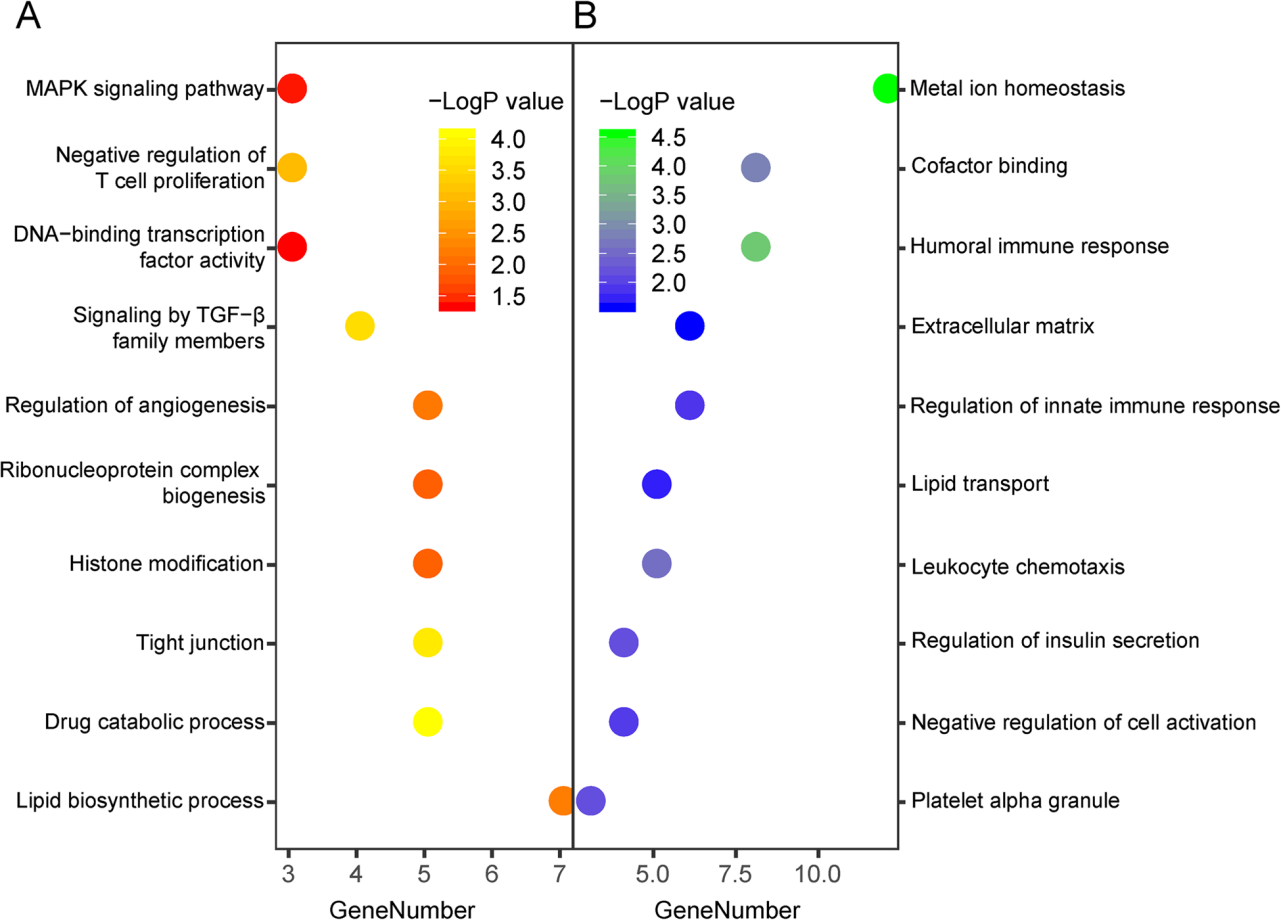
Pathway enrichment analysis of DEGs. Significantly altered pathways were displayed in dot plots. The X-axis represents the number of DEGs which were included in the pathways, and the color of dots represents the P value of the pathways. **A** Up-regulated pathways. **B** Down-regulated pathways

In order to figure out the alteration in TSC-RAML transcriptome, pathway analysis was performed using the Metascape software. The lipid biosynthetic process, signaling by TGF-β family members, and the MAPK pathway were found to be up-regulated in the TSC-RAML. The humoral immune response and the leukocyte chemotaxis pathway were found to be down-regulated (Fig. 3).

### Estimation of microenvironment cell populations

**Fig. 4:**
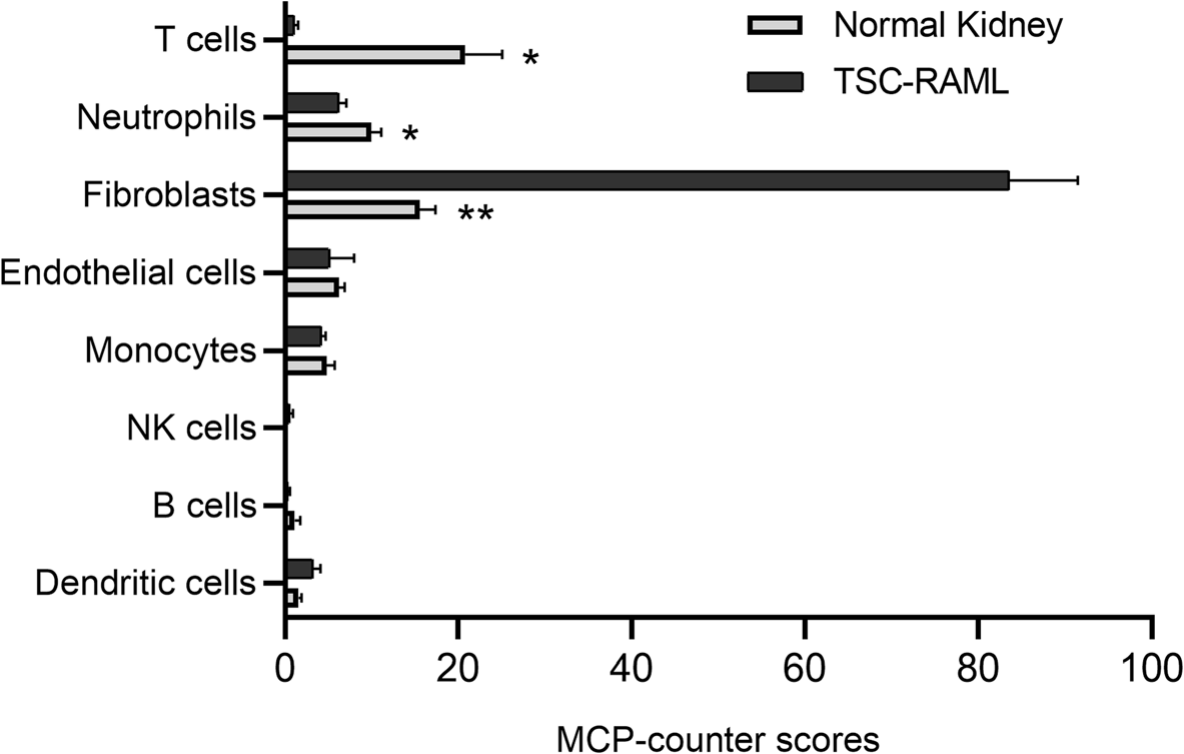
Estimated microenvironment cell populations. Estimated microenvironment cell populations were displayed with histograms. The X-axis represents MCP-counter scores of each microenvironment cell population. *P < 0.05; **P < 0.01

Microenvironment cell populations were estimated using the MCP-counter package of R software. Fibroblasts were found to be remarkably enriched in the TSC-RAML microenvironment (P = 0.003), while the T cells and neutrophils were found to be reduced in the TSC-RAML microenvironment (P = 0.015 and 0.013, respectively) (Fig. 4). No statistical alteration was found in NK cells, B cells, macrophages, and endothelial cells.

### Construction of ceRNA regulatory network

**Fig. 5:**
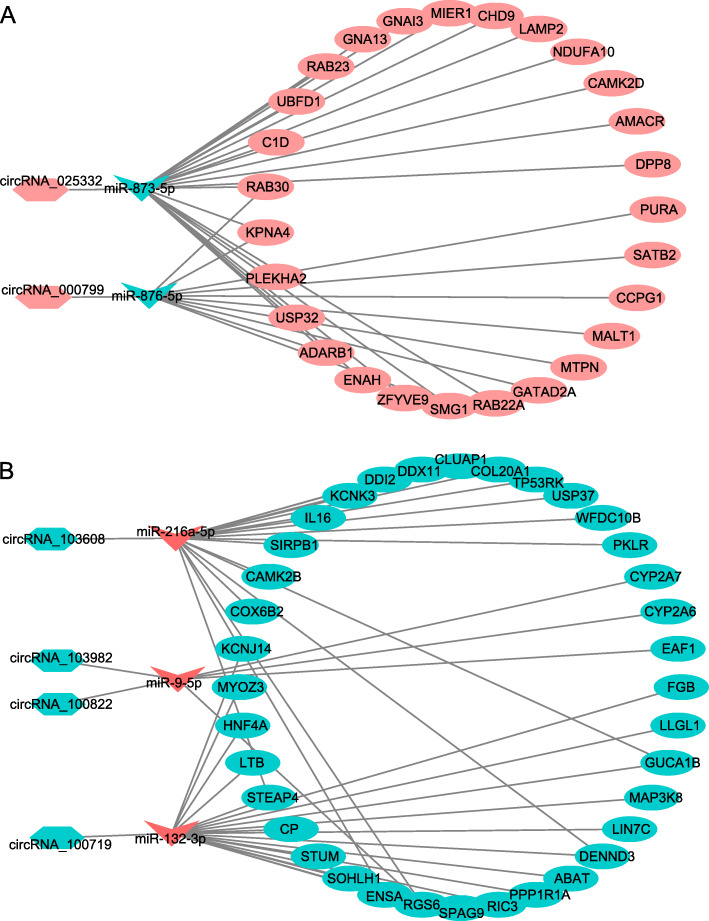
ceRNA regulatory network. A total of 6 DECs and 63DEGs were filtered into the network complex. The lines represented the regulatory relationship among these non-coding RNA and mRNAs. **A** ceRNA regulatory network constructed with down-regulated DECs, up-regulated DEmiRNAs, and down-regulated DEGs. **B** ceRNA regulatory network constructed with up-regulated DECs, down-regulated DEmiRNAs, and up-regulated DEGs

A ceRNA regulatory network was constructed using our circRNA expression profile data and transcriptome data, as well as the miRNA expression profile data reported in our previous article. All DECs in the ceRNA network were further validated by q-PCR, and the differential expression of 2 up-regulated DECs (circRNA_025332 and circRNA_000799) and 4 down-regulated DECs (circRNA_103608, circRNA_103982, circRNA_100822, and circRNA_100719) were confirmed (Fig. 1D). The final ceRNA regulatory network included 2 up-regulated DECs, 4 down-regulated DECs, 3 up-regulated miRNAs, 2 down-regulated miRNAs, and 63 mRNAs. The up-regulated DECs and down-regulated DECs were displayed separately using the Cytoscape software (Fig. 5).

### Correlation between the cell populations and DECs

**Fig. 6:**
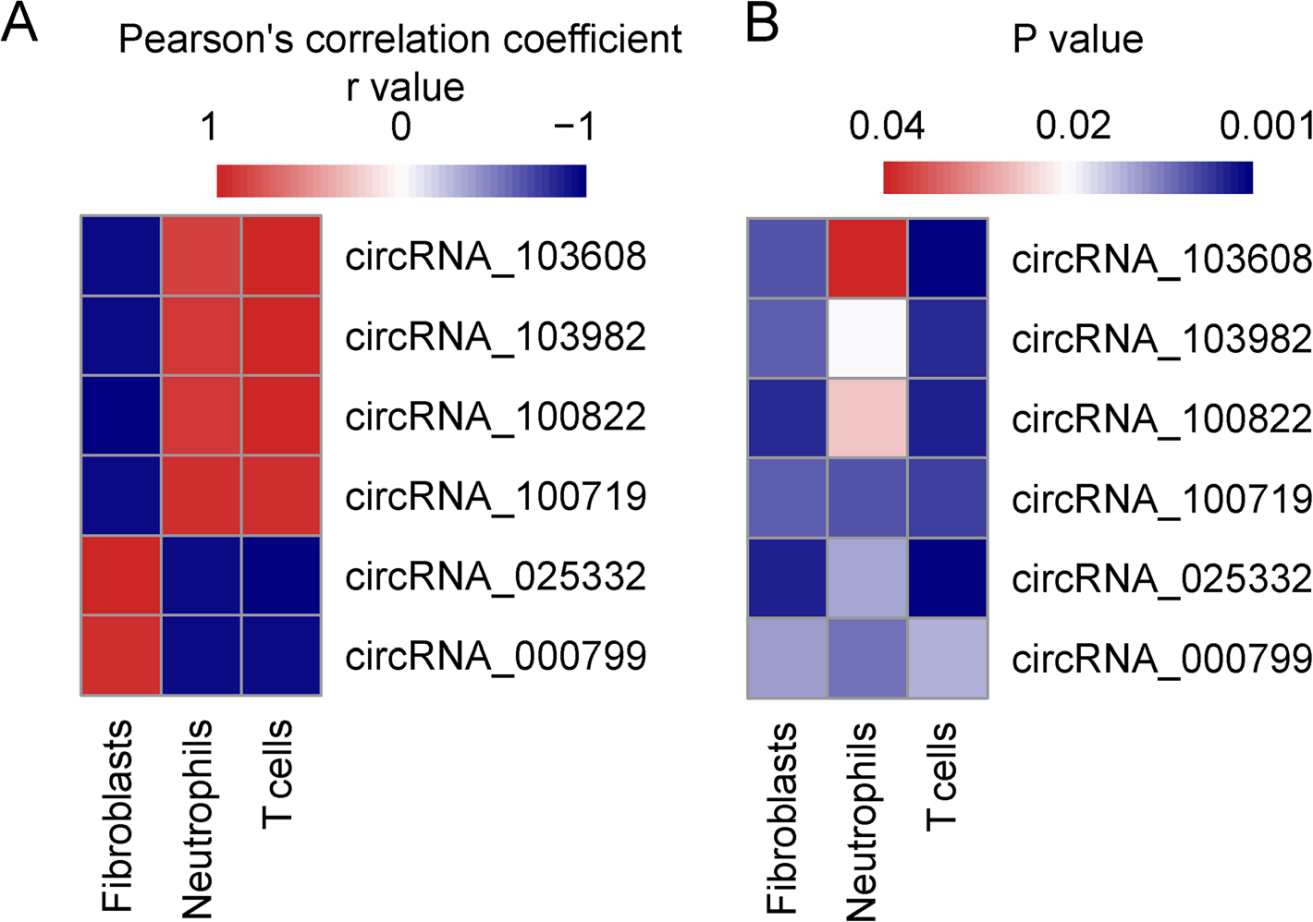
Correlation heatmaps between the cell populations and DECs. **A** The Pearson’s correlation coefficient r values between the cell populations and DECs were displayed with heatmap. **B** The significance of the Pearson’s correlation between the cell populations and DECs were displayed with heatmap

The correlation between all DECs in the ceRNA regulatory network and all cell types with significant population alteration were calculated using the R software. The expression of circRNA_000799 and circRNA_025332 were found to be positively correlated to the infiltration of fibroblast (Pearson’s correlation coefficient r value = 0.905 and 0.956, respectively; P < 0.05). The expression of the six other DECs were found to be positively correlated with the infiltration of T cells and neutrophils (Fig. 6).

## Discussions

Tuberous sclerosis complex (TSC) is a rare autosomal dominant disease caused by the mutation in the TSC1 gene or TSC2 gene. Under normal conditions, the hematin and tuberin protein, which are coded by the TSC1 gene and TSC2 gene, form a complex and inactive the Rheb (Ras-homolog enriched in brain) via the GTPase activating protein (GAP) domain of TSC2 [18]. Rheb is an activator of the protein kinase activity of mTORC1 (mammalian target of rapamycin complex 1), which is a central regulator in the mTOR pathway [19]. The nonsense or missense mutations in TSC1 or TSC2 gene could release the inhibition on Rheb activity by the hamartin-tuberin complex and up-regulate the mTOR pathway by activation of the mTORC1 [20].

In this study, GSVA analysis showed that the mTOR pathway is activated in TSC-RAML, which is consistent with the mechanism of TSC pathogenesis. Further, pathway analysis showed that genes included in the lipid biosynthetic process are significantly up-regulated in TSC-RAML samples (Fig. 3). The mTOR pathway was known as a key regulator of lipid metabolism. The mTORC1 regulates the activation of several lipid metabolism-related transcription factors, such as SREBP1 (sterol regulatory element-binding protein 1), PPARα (peroxisome proliferator-activated receptor α), and Lipin1 [21]. In our previous clinical study, we found that lipid-rich lesions could be found in most TSC-RAMLs, and TSC-RAMLs with more lipid-rich lesions showed less tumor reduction in response to mTOR inhibitor treatment [22]. Targeting lipid metabolism was thought to be a promising strategy for the treatment of TSC-RAMLs [20]. However, retrospective and prospective clinical studies showed that TSC patients cannot benefit from simvastatin, which is a classic lipid-metabolism regulator targeting HMG-CoA reductase [23, 24]. In our study, we found that circRNA_025332 showed significantly higher expression in TSC-RAML. The up-regulated circRNA_025332 may sponge for miRNA873-5p and increase the expression of AMACR, LAMP2, PLEKHA2, and CHD9, which was included in the metabolism of lipid pathway of the Reactome Gene Sets. The AMACR gene encodes an enzyme which regulates β-oxidation of branched chain lipids in peroxisomes and mitochondria and promotes chiral reversal of 2-methyl acids [25]. The LAMP2 gene encodes the lysosomal-associated membrane protein 2, which facilitates the transport of cholesterol [26]. Therefore, circRNAs may play an important role in the regulation of lipid metabolism in TSC-RAML, and circRNA_025332 may be a potential target for lipid metabolism targeting therapy.

In the present study, we found that the expression of the only two up-regulated DECs in the ceRNA regulatory network, circRNA_000799 and circRNA_025332, were positively correlated to the infiltration of fibroblast (Pearson’s correlation coefficient r value > 0.9) (Fig. 6). These results suggest that the up-regulation of circRNA_000799 and circRNA_025332 may be closely related to the infiltration of the fibroblasts. In addition, compared with normal kidney tissue, the estimated infiltration of fibroblasts remarkably increased in TSC-RAML (Fig. 4). Fibroblast is a major cell group in the TSC-RAML microenvironment. Darling et al. [27] studied the TSC skin hamartomas and found more enhanced proliferation and mTOR activation in fibroblast-like cells in skin lesions of TSC patients. It is generally agreed that fibroblast is a key progenitor of adipocyte. 3T3-L1 cells, a mouse embryotic fibroblast cell line, could transform into adipocytes under high glucose and insulin environment [28]. Our previous study showed that the lipid component is a common compartment of TSC-RAML, and it is resistant to the mTOR inhibitor treatment. Therefore, further study into the regulation of fibroblast metabolism by circRNAs is needed to find possible treatment target for mTOR-resistant TSC-RAMLs.

T cells are initiators of anti-tumor immune response and major targets of immunotherapy. In vivo studies showed that loss of TSC1 function can lead to the apoptosis of T cells and remarkably suppress the infection-specific immune responses of T cells [29]. In previous studies, immunohistochemistry staining showed that RAMLs contain elevated numbers of T cells that exhibit markers of T cell exhaustion [20]. In our pathway analysis, the genes involved in the humoral immune response and regulation of innate immune response were also down-regulated (Fig. 3). Recently, in vitro study showed that anti-PD1 and anti-CTLA4 caused prominent tumor reduction in allograft TSC tumors [30]. Therefore, targeting the PD1 and CTLA4 in T cells is a potential treatment for TSC-RAML. However, according to our transcriptome data, the estimated T cells and neutrophils in the TSC-RAML microenvironment significantly decreased (Fig. 4). Besides, our pathway analysis showed that genes involved in the negative regulation of the T cell population are up-regulated, while genes involved in the leukocyte chemotaxis are down-regulated (Fig. 3). As the lack of target cell may reduce the effect of immunology therapy, further studies are needed to investigate the relationship between the effect of immunology therapy and T cell population.

We also find that several genes included in the regulation of angiogenesis pathway are up-regulated in the TSC-RAMLs. The growth of tumors requires rapid angiogenesis to acquire nutrients and oxygen and evacuate metabolic wastes and carbon dioxide. The ability of inducing angiogenesis is a general hallmark of tumor [31]. TSC2-null cells originated from the renal tubular epithelium showed remarkably high VEGF-D expression compared with the renal tubular epithelial M1 cells with normal TSC2 gene [32]. The effect of targeting angiogenesis on TSC-RAML is still unclear, but targeting VEGFR signaling with axitinib could attenuate the growth of TSC2-null lung lesion in animal models [32].

The sample size of this study is relatively small, which is a major limitation of this study, but TSC is a rare disease, and TSC-RAML with surgical indication is even rarer. In the TuberOus SClerosis registry to increase disease Awareness (TOSCA), which is the largest multi-center TSC registry study, only 88 TSC-RAML surgery were documented, including 67 surgeries before the diagnosis of TSC [33]. The collection of TSC-RAML is extremely hard even in big medical centers, given that surgery is not the first-line treatment for TSC-RAML. Our study is the first study reporting on the high-throughput analysis of TSC-RAML circRNAs. Based on the results of clustering analysis and the typical clinical and genetic characteristics of the patients, we believe these data can represent the status of TSC-RAML and this study may help to improve our limited knowledge of TSC-RAML. However, studies with bigger sample size are needed to further study the regulatory effect of circRNAs in TSC-RAML.

## Conclusion

In conclusion, this is the first study to explore the expression profiles of circRNAs and mRNAs in TSC-RAMLs. We established a ceRNA regulatory network which included 6 DECs, 5 miRNAs, and 63 mRNAs. Lipid biosynthetic process is significantly up-regulated in TSC-RAML, and circRNA_025332 may regulate the lipid metabolism by sponging for miRNA873-5p. Fibroblasts are enriched in TSC-RAMLs, and the lipid metabolism in fibroblasts is a potential treatment target for TSC-RAML. Studies into the T cell-related immune response and the angiogenesis may also provide new treatment targets for TSC-RAMLs.

## Supplementary Information

**Additional file 1: Table S1** Clinical features of the patients.

**Additional file 2: Table S2** The sequences of primer used in this study.

## Data Availability

The datasets presented in this study can be found in China National Center for Bioinformation (https://bigd.big.ac.cn/) with the accession number PRJCA003742.
