# Supplementary material for: High-throughput screening of circRNAs reveals novel mechanisms of tuberous sclerosis complex-related renal angiomyolipoma
Source: Hum Genomics. 2021 Jul 9;15:43. doi: 10.1186/s40246-021-00344-1 (PMC8272316; doi:10.1186/s40246-021-00344-1)
Supplement: Supplementary file 1 — Additional file 1: Table S1 Clinical features of the patients. [file 40246_2021_344_MOESM1_ESM.docx]

|  | Age | Gender | Disease | Sample type | Major TSC feature | Germline TSC  mutation | Maximum tumor diameter(cm) |
| --- | --- | --- | --- | --- | --- | --- | --- |
| TSC1 | 35 | Female | TSC-RAML | Tumor tissue | Bilateral RAML;  Facial angifibroma;  Subependymal nodule. | TSC2; NM_000548; c.3683_3684 insG | 8.9 |
| TSC2 | 43 | Male | TSC-RAML | Tumor tissue | Bilateral RAML;  Facial angifibroma;  Forehead plaque;  Ungual fibroma | TSC2; NM_000548; c.4129 C>T | 4.8 |
| TSC3 | 40 | Female | TSC-RAML | Tumor tissue | Bilateral RAML;  LAM;  Facial angifibroma | TSC1; NM_000368; c.2227C>T | 8.4 |
| TSC4 | 33 | Female | TSC-RAML | Tumor tissue | Bilateral RAML;  LAM;  Facial angifibroma;  Shagreen patch | TSC1; NM_000368; c.733C>T | 8 |
| Control1 | 47 | Female | S-RAML | Kidney tissue | None | None | 2.9 |
| Control2 | 46 | Female | S-RAML | Kidney tissue | None | None | 3 |
| Control3 | 31 | Male | S-RAML | Kidney tissue | None | None | 1.8 |

**Table S1** **Clinical features of the patients**

S-RAML: Sporadic RAML

LAM：lymphangiomyoma
