# Supplementary material for: High-throughput screening of circRNAs reveals novel mechanisms of tuberous sclerosis complex-related renal angiomyolipoma
Source: Hum Genomics. 2021 Jul 9;15:43. doi: 10.1186/s40246-021-00344-1 (PMC8272316; doi:10.1186/s40246-021-00344-1)
Supplement: Supplementary file 2 — Additional file 2: Table S2 The sequences of primer used in this study. [file 40246_2021_344_MOESM2_ESM.docx]

Table S2 The sequences of primer used in this study

| ProbeID | circBase ID | F (5’-3‘) | R (5’-3‘) |
| --- | --- | --- | --- |
| hsa_circRNA_103608 | hsa_circ_0069249 | AAGCATCTCAAGGAAGGAGGC | CTGCTCAGTGAACGAGTCTGT |
| hsa_circRNA_103982 | hsa_circ_0006087 | ACCTTGGCTGGGAAAATACAT | CGCTGTGTCTGAGTTCTTGA |
| hsa_circRNA_100822 | hsa_circ_0022168 | GGCTACTAATGAGGGTTACGGG | GAACTGCAGAGAACACGGCT |
| hsa_circRNA_100244 | hsa_circ_0000075 | AAATGCAGGCCCCGAAACTT | ACCTCTCTGGTTTCTGTTCTCC |
| hsa_circRNA_104737 | hsa_circ_0086418 | AGTTCGGAACCAGAACGACT | TGACTGTTGGGACATTCTGA |
| hsa_circRNA_100719 | hsa_circ_0020390 | AGGCAGCTGATGAAAGTCGAT | AGATGTGCACAGTGTCTCCG |
| hsa_circRNA_025332 | hsa_circ_0025332 | TACCTGCAAGCAAGGCTACC | GGAAGTCACCTCCCCAAATAACT |
| hsa_circRNA_000799 | hsa_circ_0000325 | AACGCAGACGAAAATGGAAAG | AGCATTGCCCTTCTATTGGTA |
